# Carvedilol Attenuates Inflammatory-Mediated Cardiotoxicity in Daunorubicin-Induced Rats

**DOI:** 10.3390/ph4030551

**Published:** 2011-03-17

**Authors:** Flori R. Sari, Wawaimuli Arozal, Kenichi Watanabe, Meilei Harima, Punniyakoti T. Veeravedu, Rajarajan A. Thandavarayan, Kenji Suzuki, Somasundaram Arumugam, Vivian Soetikno, Makoto Kodama

**Affiliations:** 1 Department of Clinical Pharmacology, Faculty of Pharmaceutical Sciences, Niigata University of Pharmacy and Applied Life Sciences, Niigata City 956-8603, Japan; 2 Department of Pharmacology, Faculty of Medicine and Health Sciences, Syarif Hidayatullah Jakarta, State Islamic University, South Jakarta 15412, Indonesia; 3 Department of Pharmacology, Faculty of Medicine, University of Indonesia, Jakarta 10430, Indonesia; 4 Department of Gastroenterology and Hepatology, Niigata University Graduate School of Medical and Dental Sciences, Niigata 951-8510, Japan; 5 First Department of Internal Medicine, Niigata University Graduate School of Medical and Dental Sciences, Niigata 951-8510, Japan

**Keywords:** daunorubicin, carvedilol, inflammation, fibrosis

## Abstract

Cardiotoxicity, which results from intense cardiac oxidative stress and inflammation, is the main limiting factor of the anthracyclines. Carvedilol, a beta blocker that is used as a multifunctional neurohormonal antagonist, has been shown to act not only as an anti-oxidant, but also as an anti-inflammatory drug. This study was designed to evaluate whether carvedilol exerts a protective role against inflammation-mediated cardiotoxicity in the daunorubicin (DNR)-induced rats. Carvedilol was administered orally to the rats every day for 6 weeks at a cumulative dose of 9 mg/kg body weight DNR. DNR significantly induced cardiac damage and worsened cardiac function as well as increased cardiac mast cell density, elevating the myocardial protein and mRNA expression levels of tumor necrosis factor-α, vascular cell adhesion molecule-1, inter-cellular adhesion molecule-1, nuclear factor kappa-B, cyclooxygenase-2, monocyte chemotactic protein -1 and interleukin -6 compared to that in the control group. Cotreatment with carvedilol significantly attenuated the myocardial protein and mRNA expression levels of these inflammatory markers, decreased cardiac mast cell density, improved histological cardiac damage and cardiac functions. In conclusion, inflammation plays a significant role in DNR-induced cardiotoxicity, and carvedilol contributes to cardioprotection against inflammation-mediated cardiotoxicity in DNR-induced rats through its anti-inflammatory mechanism.

## 1. Introduction

Anthracyclines are a group of antibiotics that are among some of the most active chemotherapeutic agents. They are highly effective against a spectrum of malignancies, including both hematological and solid tumors (lymphoma, gastric cancer, small cell lung cancer, sarcoma, and breast cancer). Some of the commonly used anthracycline antibiotics include doxorubicin, daunorubicin (DNR), and epirubicin [1]. However, the clinical use of anthracyclines is largely limited by the adverse reactions accompanying their use. Besides their reversible and manageable adverse effects (e.g., nausea, myelosuppression, *etc.*), severe complications can also occur, such as carditoxicity, including congestive heart failure [2], increased interstitial myocardial fibrosis, and myocarditis/pericarditis [3]. These cumulative myocardial toxicities prevent the use of anthracyclines at the maximum myelotoxic doses for the optimal number of the courses [4].

The mechanism of anthracycline-induced cardiomyopathy is largely unknown, but a solid body of evidence indicates that oxidative stress and cardiac inflammation are involved. In patients that have undergone epirubicin treatment, early contractility impairment was significantly associated with high levels of reactive oxygen species (ROS) and markers of inflammation including interleukin (IL)-6 and its soluble receptor (sIL-6R) [5]. Earlier studies have illustrated that doxorubicin causes inflammatory reactions in the vicinity of heart tissues where it was found to increase the incidence of thrombus formation in the atria of mice [6]. *In vitro* data showed that doxorubicin affected both the viability and neutrophil adhesion of endothelial cells with clinically achievable concentrations [7]. It also induces inflammatory effects in the vasculature and the myocardium and elevates the levels of proinflammatory cytokines [7]. These inflammatory reactions may be mediated through the elevation of the expression levels of nuclear factor kappa-light-chain-enhancer of activated B cells (NF-κB) [8], vascular cell adhesion molecule (VCAM)-1 [6], and tumor necrosis factor (TNF)-α [9]. We have previously reported that the expression of Fas ligand (Fas-L) was significantly increased in DNR-induced rats, which also displayed significant increases in apoptosis and cardiotoxicity [10]. Fas-L itself has been well-characterized as a death factor and plays an important role as an upstream molecule of many inflammatory factors that induce the expression of a variety of inflammatory cytokines *in vivo* [11]. Thus, previous studies have indicated that inflammatory reactions are involved in DNR-induced cardiotoxicity. However, further experimental studies are needed to verify and to elucidate these possibilities.

**Figure 1:**
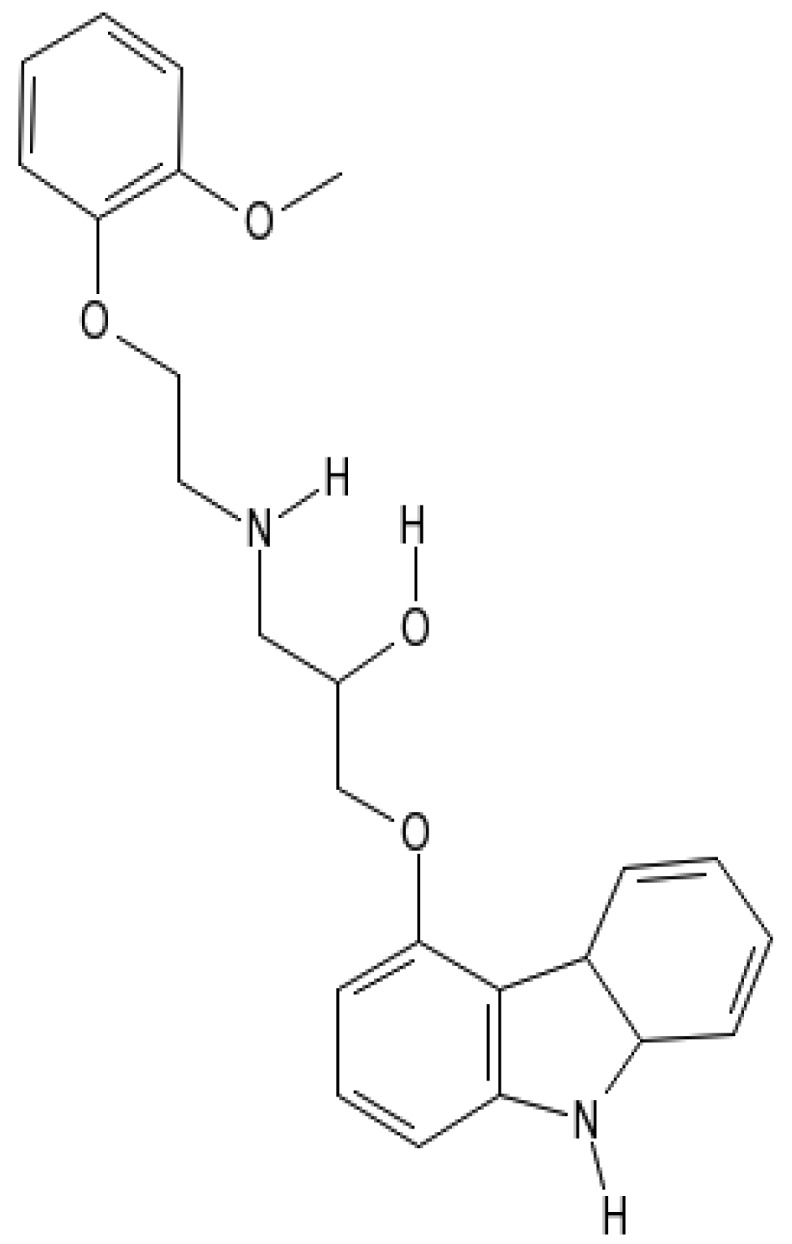
Structure of carvedilol.

Carvedilol (Coreg™, Dilatrend™, Eucardic™, Carloc™, (±)-[3-(9*H*-carbazol-4-yloxy)-2-hydroxypropyl][2-(2-methoxyphenoxy)ethyl]amine, Figure 1), a multifunctional neurohormonal antagonist, has been shown to provide greater benefit than traditional β-blockers because of its antioxidant actions, which act in synergy with its nonspecific β- and α1- blocking effects [12]. Furthermore, carvedilol has been reported to have an anti-inflammatory effect by inhibiting IL-10 and IL-18 in patients with heart failure [13] and by inhibiting the expressions of VCAM-1, inter-cellular adhesion molecule (ICAM)-1, and IL-8 via NF-κB in the human endothelial cells [14]. Accumulating evidences have shown that carvedilol protects against anthracycline-induced cardiotoxicity through its anti-oxidant properties [15-17]. However, whether carvedilol exerts a protective role against inflammation-mediated cardiotoxicity in DNR-induced rats should be further studied. Thus, the present study was conducted to assess the possible role of inflammation-mediated cardiotoxicity in DNR-induced rats and to verify the anti-inflammatory effect of carvedilol against DNR-induced cardiotoxicity. In this study, we investigated the changes in left ventricular (LV) function, histopathology, and the levels of inflammatory markers in rats given DNR and DNR in combination with carvedilol.

## 2. Experimental

### 2.1. Drugs and Chemicals

Unless otherwise stated all reagents were of analytical grade and purchased from Sigma (Tokyo, Japan). DNR was kindly donated by Meiji Seika Kaisha Ltd, Tokyo, Japan. Carvedilol was donated by Daichi-Sankyo Pharmaceutical (Tokyo, Japan).

### 2.2. Animals and Medication

Eight-week-old male Sprague-Dawley rats were obtained from Charles River Japan Inc. (Kanagawa, Japan). On day 0, each animal received a single intravenous injection of DNR at a dose of 3 mg/kg (i.v.). The drug was administered in three equal injections at 48 h intervals for a period of a week to achieve an accumulative dose of 9 mg/kg, which is well documented to achieve cardiotoxicity and nephrotoxicity [10,18,19]. Twenty DNR-treated rats were randomly divided into two groups and received oral administration of carvedilol (30 mg/kg/day; group Carv; n = 10) or vehicle (group DNR; n = 10). Age-matched rats were injected with corresponding volumes of 0.9% NaCl and used as a control (group Control; n = 4). The dose of carvedilol was chosen on the basis of our preliminary study (data not shown). Administration of carvedilol was started on the same day as DNR administration and continued for 5 additional weeks after cessation of DNR administration (6 weeks total period). On day 41, the body weight (BW) was measured. Throughout the study, all animals were cared for in accordance with the guidelines of our institute and the Guide for Care and Use of Laboratory Animals published by the US National Institutes of Health.

### 2.3. Hemodynamic and Echocardiographic Study

After the end of the study period (6 weeks), rats were anesthetized with 2% halothane in O_2_ and subjected to surgical procedures to measure hemodynamic parameters. After the instrumentation, the concentration of halothane was reduced to 0.5% to record steady-state hemodynamic data. Hemodynamic parameters such as mean blood pressure (MBP), peak left ventricular pressure (LVP), LV end-diastolic pressure (LVEDP), and the rate of intra-ventricular pressure rise and decline (± dP/dt) were recorded as previously described [20].

Two-dimensional echocardiographic studies were performed under 0.5% halothane anesthesia using an echocardiographic machine equipped with a 7.5-MHz transducer (SSD-5500; Aloka, Tokyo, Japan). M-mode tracings were recorded from the epicardial surface of the right ventricle; the short axis view of the LV was recorded to measure the LV dimension in diastole (LVDd) and LV dimension in systole (LVDs). LV fractional shortening (FS) and ejection fraction (EF) were calculated and expressed as percentages. The study was performed in a blinded manner.

### 2.4. Histopathological Analysis

After the measurement of echocardiographic parameters, hearts were excised and weighed immediately (HW), and their ratios to BW (HW/BW) were calculated. Half of each heart was immediately snap-frozen in liquid nitrogen for subsequent protein extraction and enzymatic assays. The remaining excised hearts were cut into about 2-mm-thick transverse slices and fixed in 10% formalin. After being embedded in paraffin, several transverse sections were obtained from the ventricle and stained with hematoxylin-eosin (HE) for histological evaluation, and also stained with Azan-Mallory to demonstrate fibrosis in cardiac tissues. The frequency and the severity of lesions in the hearts were assessed semi-quantitatively as previously reported [21,22] by light microscopy using a scale where score 0, normal; 1, mild; 2, moderate; and 3, severe. The scoring criteria for myocardial lesions included the degree of myocyte vacuolization with respect to size and the number of vacuoles, degree of fibrosis, myocardial degeneration and interstitial edema and loss of myofibrils.

Histochemical staining with toluidine blue was performed to identify mast cells. For toluidine blue staining, slides of paraffinized sections of the mid-ventricles were dewaxed, rehydrated and incubated with 0.05% w/v toluidine blue for 30 min followed by counterstaining with 0.01% w/v eosin for 15 min. Mast cell density was quantified by counting the number of toluidine-blue positive mast cells per field (200×) in the whole heart section and shown as a percentage.

### 2.5. Protein Analysis by Western Blotting

Protein lysate was prepared from heart tissues as described previously [23]. The total protein concentration in samples was measured by the bicinchoninic acid method. For the determination of protein levels of TNF-α, VCAM-1, ICAM-1, cyclooxygenase (COX)-2, glyceraldehyde 3-phosphate dehydrogenase (GAPDH) (Santa Cruz Biotechnology Inc., CA, USA) and NF-κB (Cell Signaling Technology, Inc., Beverly, MA, USA) equal amounts of protein extracts (30 μg) were separated by sodium dodecyl sulfate (SDS) polyacrylamide gel electrophoresis (Bio-Rad, Richmond, CA, USA) and transferred electrophoretically to nitrocellulose membranes. Membranes were blocked with 5% non-fat dry milk in Tris-buffered saline Tween (20 mM Tris, pH 7.6, 137 mM NaCl, and 0.1% Tween 20). All antibodies were used at a dilution of 1:1000. The membrane was incubated overnight at 4 °C with the primary antibody, and the bound antibody was visualized using the respective horseradish peroxidase-conjugated secondary antibodies (Santa Cruz Biotechnology Inc.) and chemiluminescence developing agents (Amersham Biosciences, Buckinghamshire, U.K.). The level of GAPDH was estimated in every sample to check for equal loading of samples. Films were scanned, and band densities were quantified with densitometric analysis using Scion Image program (Epson GT-X700, Tokyo, Japan). All values were normalized by setting the density of control samples as 1.0.

### 2.6. Quantitative Real-Time Polymerase Chain Reaction (RT-PCR) to Detect Monocyte Chemotactic Protein (MCP)-1 and IL-6

Total RNA was extracted from heart tissues with Trizol (Gibco BRL) in accordance with the standard protocol and reverse-transcribed. Thereafter, cDNA was amplified using the ABI 7700 sequence-detector system (Applied Biosystems, Foster City, CA, USA) with a set of primers and probes corresponding to MCP-1, IL-6 and GAPDH as previously described [24].

### 2.7. Statistical Analysis

Data are presented as mean ± SEM and were analyzed using one-way analysis of variance (ANOVA) followed by Tukey or Bonferroni methods for post hoc analysis and two-tailed t-test when appropriate. A value of *p* < 0.05 was considered statistically significant. For statistical analysis GraphPad Prism 5 software (GraphPad Software, San Diego, CA, USA) was used.

## 3. Results and Discussion

### 3.1. Effects of Carvedilol on BW and Survival Rate

As depicted in Table 1, BW was significantly decreased in the DNR and carvedilol groups of rats compared with the control group. Carvedilol treatment tended to increase the BW compared with that in the DNR group, although the effect did not attain statistical significance. The mortality rate was higher in the DNR group than that of the control group. Conversely, the mortality rate was lower in carvedilol group than that in the DNR group. In brief, five of 10 (50%) and two of 10 (20%) rats in the DNR and carvedilol groups, respectively, died between days 28 and 42 (Table 1). Furthermore, in prematurely dead animals in both DNR and carvedilol groups, a necropsy examination revealed marked sign of blood congestion such as hydrothorax and ascites. None of the rats in the control group died.

### 3.2. Effect of Carvedilol on Daunorubicin-Induced Cardiotoxicity

**Figure 2:**
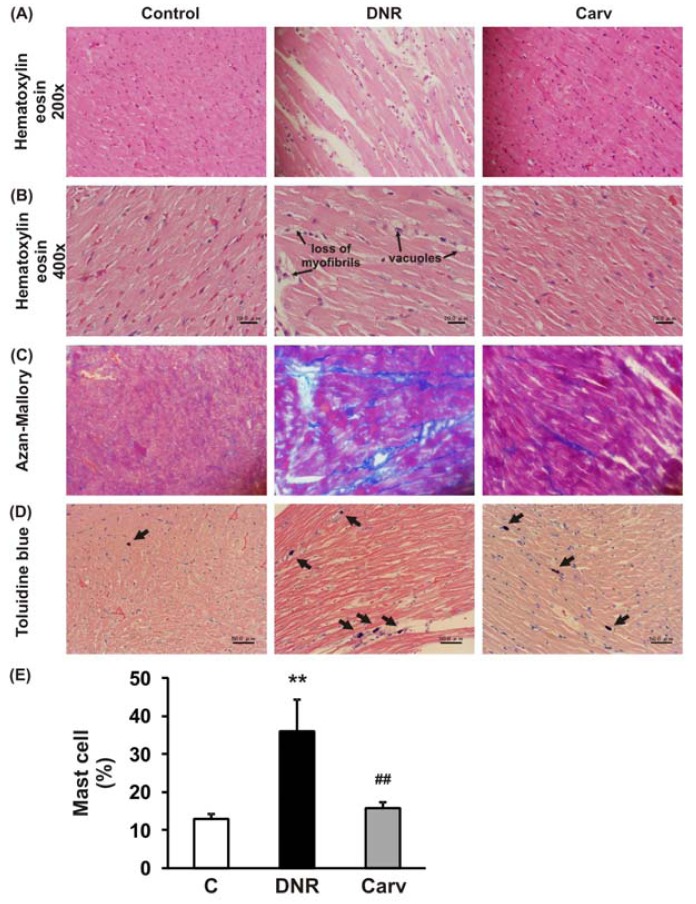
**A,B**, Hematoxylin and eosin staining of the cross-sectional tissue slices of hearts depicting vacuolation and degeneration of cardiac fibers at the magnification of 200× and 400×, respectively. **C**, Azan-Mallory staining for fibrosis of the cross-sectional tissue slices of hearts. Fibrosis is indicated by the blue area as opposed to the red myocardium at the magnification of 200×. **D**, Toluidine blue staining for mast cell of the cross-sectional tissue slices of hearts. Metachromatic staining of mast cell granules was handy to identify these cells. Toluidine blue-positive mast cell was demonstrated in purple (shown by arrows) at the magnification of 200×. **E**, Quantification of mast cell staining. In E, the white, black and grey bars, respectively, represent the control (age-matched normal rats), DNR (DNR-treated rats administered with vehicle) and the Carv (DNR-treated rats administered with carvedilol (30 mg/kg/day)) group. Each bar represents mean ± S.E.M. **p < 0.01 *vs.* group Control; ^##^*p* < 0.01 *vs.* group DNR.

The definition of anthracycline-induced cardiotoxicity has expanded from the clinical events of cardiac failure to include a wide spectrum of predefined laboratory values, even in asymptomatic patients [1]. These include histological changes in the cardiomyocytes [25] and changes in LVEF [26]. As depicted in Table 2 and Figure 2A, 2B, HE staining showed that DNR induced significant cardiac damage as recognized by the presence of marked interstitial edema, focal subendocardial fibrosis, perinuclear vacuolation, and disorganization and degeneration of the myocardium compared to the control group. The morphological changes in the myocardia of the DNR-induced animals were also characterized by focal myocardial interstitial fibrosis of varying intensity [27]. The Azan-Mallory staining shown in Figure 2C demonstrated significant interstitial myocardial fibrosis in the DNR group compared with the control group. Carvedilol treatment significantly improved these lesions (Figure 2 and Table 2).

Our present study indicated that the cumulative administration of DNR resulted in cardiotoxicity as evidenced by worsening myocardial function. LVEDP was significantly higher (10.8 ± 0.2 *vs.* 7 ± 1 mmHg, *p* < 0.05) and LVP and ± dP/dt were significantly lower in the DNR group compared with the control group (105 ± 7 *vs.* 125.3 mmHg, *p* < 0.05; and 4635 ± 351 *vs.* 7123 ± 544 mmHg/s, *p* < 0.05; 3906 ± 329 *vs.* 7851 ± 656 mmHg/s, *p* < 0.05, respectively), indicating that systolic and diastolic dysfunction occurred in the DNR rats. Carvedilol treatment improved this myocardial dysfunction by significantly reducing LVEDP (8.2 ± 1.2 vs. 10.8 ± 0.2 mmHg, *p* < 0.05) and significantly elevating in the LVP and + dP/dt (120.5 ± 11 *vs.* 105 ± 7 mmHg, *p* < 0.05; and 6229 ± 581 *vs.* 4635 ± 351 mmHg/s, *p* < 0.05, respectively) compared with those in the DNR group (Table 3). Echocardiographic data showed that LV systolic function, as assessed by FS and EF was significantly reduced in the DNR group compared with that in the control group (28.4 ± 1.1 *vs.* 43.8 ± 1.5%, *p* < 0.05; and 59.4 ± 1.4 *vs.* 79.9 ± 1.8%, *p* < 0.05, respectively). Carvedilol significantly attenuated the reductions in both FS and EF compared with the DNR group (38.8 ± 3.5 *vs.* 28.4 ± 1.1%, *p* < 0.05; and 73.6 ± 4.4 *vs.* 59.4 ± 1.4%, *p* < 0.05, respectively) (Table 3).

### 3.3. Effect of Carvedilol on Inflammatory-Mediated Cardiotoxicity

The mechanism of anthracycline-induced cardiomyopathy have been the subject of considerable controversy, nevertheless the iron-mediated formation of reactive oxygen species and promotion of myocardial oxidative stress remains by the far the most frequently proposed mechanism. Similarly, there is a strong association between oxidative stress and the cardiac inflammatory response, including cytokine release after doxorubicin treatment [28]. In the clinical setting, it would be difficult to measure the inflammatory process in anthracycline-based therapy in the context where stimulation has activated the inflammatory process. In patients receiving chronic administration of anthracycline, endomyocardial biopsy data showed no inflammatory cells were found in the endocardial zone [29]. On the contrary, Mills *et al.* found that markers on inflammation (*i.e* VEGF, sICAM-1, sP-selectin, vWf) were significantly increased after four-3 weeks treatment of anthracycline compared with before treatment in the breast cancer patient [30]. Caddedu *et al.* found that IL-6 and TNF-α were significantly up-regulated in the patient received epirubicin treatment [31]. These results have shown the possibility that inflammation process appeared from the progression of the primary disease. This matter has become the limitation of our study since carvedilol was given in the same timing with the DNR. Therefore, we cannot evaluate further the inflammation process at the timing “after DNR” but “before carvedilol”. However, Zhu *et al.* demonstrated further that IL-1 family is closely connected with doxorubicin-induced cardiotoxicity. At day seven after doxorubicin treatment, IL-1β and IL-1Ra were both highly induced after different dosages of doxorubicin treatments. In the separated experiments, they found that administration of recombinant human interleukin-1 receptor antagonist (rhIL-1Ra) prevents heart damage, protects cardiac function loss and decreases cardiomyocyte apoptosis in doxorubicin-treated mice [32].

**Figure 3:**
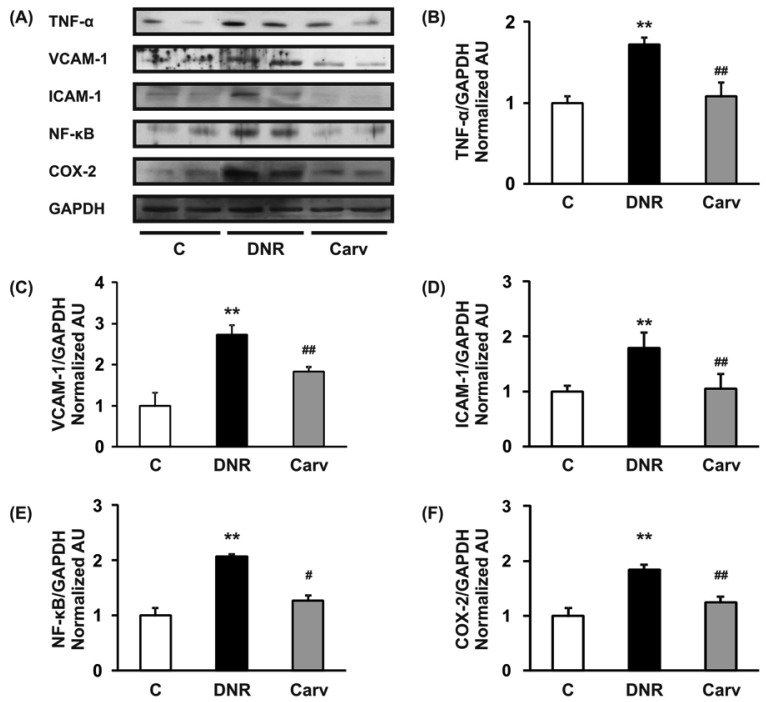
A, Western immunoblots of myocardial TNF-α, VCAM-1, ICAM-1, NF-κB and COX-2 are showing two representative experiments out of four. All bands were normalized against GAPDH. B-F, Densitometric data of protein analysis. In B-F, the white, black and grey bars, respectively, represent the control (age-matched normal rats), DNR (DNR-treated rats administered with vehicle) and the Carv (DNR-treated rats administered with carvedilol (30 mg/kg/day)) group. Each bar represents mean ± S.E.M. **p < 0.01 *vs.* group Control; ^#^*p* < 0.05 and ^##^*p* < 0.01 *vs.* group DNR.

We have previously reported that the ROS level and Fas-L expression level were significantly up-regulated in the DNR-induced rats, which also displayed significant increases in apoptosis and cardiotoxicity [10,18]. Even though Fas-L has been well characterized as a death factor, it also plays a pivotal role as an upstream inflammatory factor that induces the expression of a variety of other inflammatory cytokines *in vivo* [11]. In mice received doxorubicin treatment, the activation of Fas signaling is also a key mediator of carditoxicity, including cardiomyocyte apoptosis and myocardial inflammation and inhibiting the Fas expression with sFas gene therapy prevents the progression of cardiotoxicity caused by Fas signaling [33]. Fas expression is strongly induced in mouse embryonic fibroblasts by the inflammatory cytokine TNF-α [34]. In this study, we analyzed the toluidine blue staining to identify mast cells and found that mast cells were significantly increased in the DNR rats compared with control rat and carvedilol treatment significantly reverses this condition (Figures 2D and E). This result suggests that inflammatory process may involve in the anthracycline-induced cardiomyopathy. As depicted in Figures 3A and B, we further found that DNR produced an increase in cardiac TNF-α expression compared with the control group. It is important to note that carvedilol treatment significantly decreased the cardiac expression of TNF-α protein compared with that in the DNR group. TNF-α has also been reported to mediate cardiac damage in the doxorubicin-induced mice [9]. In accordance with our result, in the clinical setting, it has been reported that carvedilol decreased the plasma levels of TNF-α in patients with ischemic dilated cardiomyopathy [35].

Our result further showed that the myocardial protein expression levels of adhesion molecules (VCAM-1 and ICAM-1) were significantly up-regulated in the DNR group compared with the control group (Figure 3A, C and D). These increases in the expression levels of adhesion molecules may be mediated through the regulation of TNF-α and the toxicity of anthracyclines. A previous study reported that TNF-α enhanced the expression of adhesion molecules on the membranes of endothelial cells [36]. Furthermore, doxorubicin has been reported to induce the expression of VCAM-1 [6]. Our result confirmed that carvedilol significantly reversed the expression of VCAM-1 and ICAM-1 compared with the DNR group (Figures 3A, C and D). These results support those of a previously published study that demonstrated that carvedilol inhibits TNF-α-induced adhesion molecule expression in human endothelial cells [14].

During the progression of cardiovascular diseases such as congestive heart failure and atherosclerosis, TNF-α may activate membrane-bound NADPH oxidase and increase intracellular ROS production, which could in turn activate redox-sensitive transcriptional pathways such as NF-κB and regulate the expression of downstream inflammatory molecules [37]. Doxorubicin has also been reported also to induce NF-κB-associated apoptosis and NF-κB-associated cytotoxicity in endothelial cells and human colon cancer HT29 cells, respectively [38,39].

**Figure 4:**
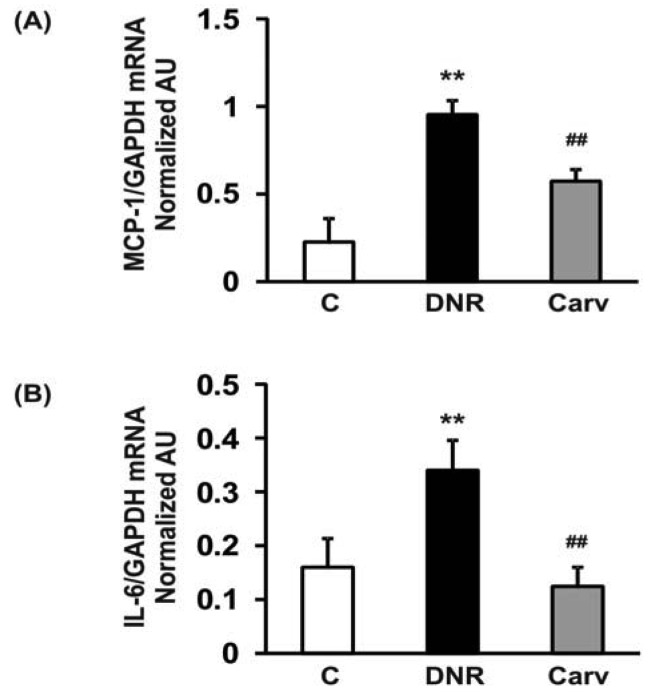
Myocardial messenger RNA expression levels of MCP-1 (A) and IL-6 (B) in DNR-rats were determined by quantitative RT-PCR. The expression level of each sample was expressed relative to the expression level of GAPDH gene. The white, black and grey bars, respectively, represent the control (age-matched normal rats), DNR (DNR-treated rats administered with vehicle) and the Carv (DNR-treated rats administered with carvedilol (30 mg/kg/day)) group. Each bar represents mean ± S.E.M. **p < 0.01 *vs.* group Control; ^##^*p* < 0.01 *vs.* group DNR.

Therefore, NF-κB may mediate anthracyclines-induced cardiotoxicity through its role in the regulation of apoptosis and the expression of downstream inflammatory molecules. In accordance with these findings, we found that DNR significantly induced the expression of transcription factors related to inflammation, e.g., NF-κB, compared to the control group. Additionally, the myocardial protein expression levels of COX-2, which is regulated by NF-κB [40], were also significantly increased in the DNR group compared with the control group. Carvedilol significantly attenuated the myocardial expression of NF-κB and COX-2 compared to that in the DNR group (Figures 3A and F). These results are reasonable since carvedilol has been shown to inhibit the expression of TNF-α-induced NF-κB in human endothelial cells [14,37]. Accumulating evidence has shown that doxorubicin plays a pivotal role in the induction of MCP-1 expression in human lung carcinoma cells [41]. Furthermore, IL-6 has been shown to be involved in doxorubicin-induced cardiotoxicity [42]. To assess whether the expression of these cytokines was induced after DNR treatment, we performed RT-PCR analysis to evaluate MCP-1 and IL-6 mRNA expression in cardiac tissues. Our results showed that the expression levels of MCP-1 and IL-6 were significantly up-regulated in the DNR group compared to those in the control group (Figures 4A and B), and carvedilol significantly down-regulated the mRNA expression of these cytokines compared to their levels in the DNR group. These results support the findings of previous studies in which carvedilol was found to exert its anti-inflammatory effects by attenuating TNF-α induced MCP-1 expression [14].

## 4. Conclusions

**Figure 5:**
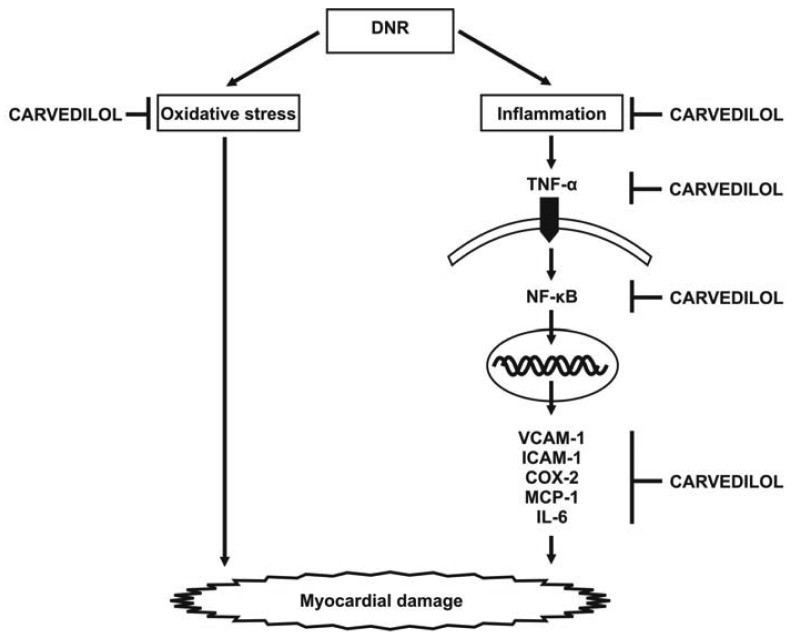
Scheme representing possible mechanism of carvedilol as an anti-inflammatory drug in DNR induced myocardial damage.

Cardiotoxicity, which may result from intense cardiac oxidative stress and inflammation, is the main limiting factor of anti-cancer therapy using anthracyclines. However, the mechanism by which inflammatory reactions elicit cardiotoxicity is largely unknown. Our recent report shows that DNR induced significant cardiac damage, as recognized by histological changes and worsening cardiac function, and significantly increased the expression levels of inflammatory cytokines and proinflammatory chemokines including TNF-α, VCAM-1, ICAM-1, NF-κB, COX-2, MCP-1 and IL-6 (Figure 5). Thus, the simultaneous induction of these inflammatory markers may augment carditoxicity in DNR-induced rats. These cytokines may produce deleterious effects on LV structure (remodeling) and endothelial function. In fact, in addition to the effects of inflammatory mediators on cardiac structure and function, there is growing evidence that the concentration of inflammatory mediators that exist in heart failure are sufficient to contribute to endothelial dysfunction [43,44]. Therefore, the lack of evaluation the serum circulating levels of cytokines and endothelium dysfunction marker such as asymmetric dimethylarginine can be considered as limitations of present study. As carvedilol is known to have anti-inflammatory effects, we used this drug to improve DNR-induced cardiotoxicity in our model. We have shown that carvedilol significantly attenuated the protein and mRNA expression levels of these markers and improved cardiac function. Based on these results, we speculate that carvedilol contributes to cardioprotection against DNR-induced cardiotoxicity through its anti-oxidant and anti-inflammation properties. Since dexrazoxane (ICRF-187) is the only clinically approved cardioprotectant against anthracycline cardiotoxicity [45], it would be of interest to combine carvedilol with dexrazoxane to evaluate the possible potential synergistic or additive effect in preventing anthracycline-induced cardiotoxicity. Further investigation is warranted to address these issues.

## Figures and Tables

**Table 1:** Changes in survival rate and HW/BW ratio after 6 weeks of treatment with carvedilol in DNR rats.

	**Control n** = **4**	**DNR n** = **10**	**Carv n** = **10**
**No. of rats died**	0	5	2
**Survival rate (%)**	100	50	80
**BW (g)**	540 ± 11.5	373 ± 7.4 *	405 ± 21
**HW (g)**	1.3 ± 0.02	1.1 ± 0.01 *	0.9 ± 0.05 *
**HW/BW (g/kg)**	2.2 ± 0.02	2.9 ± 0.05	2.4 ± 0.02

Results are presented as the mean ± SEM. BW, body weight; HW, heart weight; HW/BW, ratio of heart weight to body weight. Group Control, aged matched normal rats; group DNR, DNR rats treated with vehicle; group Carv, DNR rats treated with carvedilol (30 mg/kg/day).

* *p* < 0.05 *vs.* group Control.

**Table 2:** Effect of carvedilol on histopatological changes in cardiac tissues after 6 weeks of treatment in DNR rats.

**Histopathological Finding**	**Control n** = **4**	**DNR n** = **5**	**Carv n** = **8**
**Cardiac tissue**			
**Myocardial fibrosis**	0.0 ± 0.0	1.5 ± 0.21*	0.4 ± 0.18^#^
**Perinuclear vacuolization**	0.0 ± 0.0	0.5 ± 0.25*	0.2 ± 0.2^#^
**Myocardial degeneration**	0.0 ± 0.0	2.75 ± 0.21*	1.4 ± 0.21^#^
**Interstitial edema**	0.0 ± 0.0	2.75 ± 0.19*	1.6 ± 0.22^#^

Results are presented as the mean ± SEM. Group Control, aged matched normal rats; group DNR, DNR rats treated with vehicle; group Carv, DNR rats treated with carvedilol (30 mg/kg/day).

* *p* < 0.05 *vs.* group Control and

# *p* < 0.05 *vs.* group DNR.

**Table 3:** Changes in hemodynamic, and echocardiographic parameters after 6 weeks of treatment with carvedilol in DNR rats.

**Parameter**	**Control n = 4**	**DNR n = 10**	**Carv n = 10**
**CVP (mmHg)**	-0.5 ± 0.04	0.44 ± 0.05	0.3 ± 0.05
**MBP (mmHg)**	96 ± 7.4	87 ± 6.4	82 ± 9.7
**LVP (mmHg)**	125.3 ± 5	105 ± 7*	120.5 ± 11^#^
**LVEDP (mmHg)**	7 ± 1	10.8 ± 0.2*	8.2 ± 1.2^#^
**+dP/dt (mmHg/s)**	7123 ± 544	4635 ± 351*	6229 ± 581^#^
**-dP/dt (mmHg/s)**	7851 ± 656	3906 ± 329*	5034 ± 516^*^
**HR (beats/min)**	362 ± 34	329 ± 11	317 ± 31
**LVDd (mm)**	6.5 ± 0.5	7.8 ± 0.1	7.05 ± 0.4
**LVDs (mm)**	4.2 ± 0.3	5.5 ± 0.2	4.9 ± 0.5
**FS (%)**	43.8 ± 1.5	28.4 ± 1.1*	38.8 ± 3.5^#^
**EF (%)**	79.9 ± 1.8	59.4 ± 1.4*	73.6 ± 4.4^#^

Results are presented as the mean ± SEM. CVP, central venous pressure; MBP, mean blood pressure; LVP, left ventricular pressure; LVEDP, left ventricular end-diastolic pressure; ±dP/dt, rate of intra-ventricular pressure rise and decline; HR, heart rate; LVDd, left ventricular dimension in diastole; LVDs, left ventricular dimension in systole; FS, fractional shortening; EF, ejection fraction; group Control, aged matched normal rats; group DNR, DNR rats treated with vehicle; group Carv, DNR rats treated with carvedilol (30 mg/kg/day).

* *p* < 0.05 *vs.* group Control and

# *p* < 0.05 *vs.* group DNR.
